# No End in Sight? A Greenwash Review and Research Agenda

**DOI:** 10.1177/10860266231168905

**Published:** 2023-05-09

**Authors:** A. Wren Montgomery, Thomas P. Lyon, Julian Barg

**Affiliations:** 1 Western University, London, Ontario, Canada; 2 University of Michigan, Ann Arbor, USA

**Keywords:** greenwash, corporate environmental communications, miscommunications, symbolic management, decoupling, rhetoric, brownwash, ESG, net zero

## Abstract

Greenwashing is more virulent than ever. A profusion of environmental, social, and governance and net zero commitments are becoming fraught with questionable and misleading claims. At the same time, we are no closer to solving the pressing environmental and social issues of our time. In this review, we seek to examine this shift and summarize changes in greenwash research into three key phases: (a) 1.0 Static Communication; (b) 2.0 Dynamic Management; and (c) 3.0 Narratives about the Future. We analyze current key areas of developing literature and point to numerous open questions for future research. Next, we go beyond much of the published work to examine emerging tactics and lay out a forward-looking agenda for future research. We also propose a model of Corporate Miscommunication, integrating various streams in greenwash research. In doing so, we seek to lay a pathway for greenwashing researchers to finally find that elusive “end” to greenwashing.

## Introduction

**Figure 1.**
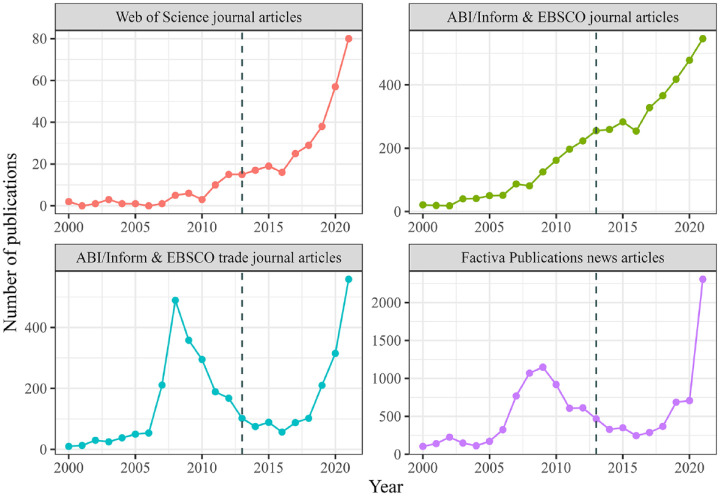
Greenwashing Trends 2000–2021. *Note.* The dotted line marks the status of the literature and publications at the time of our last review (Lyon & Montgomery, 2015). While Web of Science constitutes the most complete database of scientific journal articles, it does not offer full-text search. Therefore, this search includes only articles in which greenwashing plays an important role and appears in the title, abstract, or keywords; those results are in the upper left panel. We also searched ABI/Inform & EBSCO for academic articles, since these two searches allow more readily for the inclusion of greenwashing anywhere in the text, although greenwashing may only play a minor role in the article; those results are in the upper right panel. We searched ABI/Inform and EBSCO for trade journal articles using the same approach; those results are in the lower left panel. Duplicates have been removed for combined searches. Finally, we searched Factiva for all news articles using the same approach; those results are in the lower right panel.

Less than a decade ago, growing stakeholder demands for transparency and eco-labeling seemed to spell the imminent demise of greenwashing. An entire book was devoted to what comes “after greenwashing” (Bowen, 2014) and two of the current authors’ own widely-cited review article foresaw the “end of greenwash” (Lyon & Montgomery, 2015). Despite our optimism, today green claims are multiplying rapidly in the corporate race toward investing using environmental, social, and governance (ESG) criteria and “net zero” carbon reduction commitments. In response, popular concern about greenwashing is soaring, with news articles on the topic expanding from under 300 annually a decade ago to well over 2,000 in 2021 (see Figure 1). U.K.-based non-profit InfluenceMap found that over 55% of ESG funds committed greenwashing in the form of exaggerated claims and 70% failed to meet their ESG targets (Quinson, 2021). Academic commentators have raised concerns that the boom in net zero commitments is largely greenwash (Black et al., 2021), while Greta Thunberg infamously called the 2021 COP26 conference a “greenwashing festival” on Twitter.

What caused this worrisome upsurge? Is greenwash, like COVID, something we must simply learn to live with rather than eradicate? Does the soaring discussion of greenwash indicate that its effects today are more pernicious than in the past, threatening our society’s ability to solve the climate crisis rather than just convincing consumers to buy products that are a bit less green than they expected? Extant reviews do not tackle these newest forms of greenwash, nor offer a forward-looking scholarly agenda to fully understand and influence this pressing issue in its current forms. These are the goals of this review. In holding a mirror up to the current greenwashing literature and examining it critically, we are of course being self-critical, as well, as one of the early and impactful review papers was our own (Lyon & Montgomery, 2015), published in this journal. Which begs the questions: why another and why now?

We believe that our prior review and synthesis, as well as those that preceded and followed it, have indeed resulted in a more cohesive body of theoretical knowledge in management and related disciplines, many of which directly seek to answer the calls for future research in that paper. Since 2015, research has become more systematic, with more sophisticated empirics, but articles have become narrower and more in-depth. As the term greenwash has exploded in popularity and has become commonly used, in many different ways our prior review has been a success as it helped to shape and connect this emerging research and conversation. However, it also had several notable shortcomings. First, our title suggested an “end” to greenwashing, which is very far from what has occurred. Second, the literature at the time did not fully understand how different forms of greenwash could be deployed simultaneously against different stakeholders. Tailored greenwashing has been facilitated by the emergence of social media “bubbles,” in which people engage with others who hold similar views. Third, we simply did not anticipate the emergence of certain forms of greenwash, such as unsupported promises for future action, which have become prevalent since our prior review.

Although several notable reviews have emerged in the years since our own (de Freitas Netto et al., 2020; Gatti et al., 2019; Nemes et al., 2022), each very useful for its own reasons, we do not feel that any one of these goes beyond reviewing the current literature to address the variety of mechanisms of deception that are now evident, nor do they offer a clear and motivating call for collective research efforts that will address the deepening crisis of greenwashing. We see the growth of greenwash as corrupting, delaying, and weakening progress toward ESG goals, net zero commitments, adoption of eco -certifications, and policy change—and thereby weakening society’s collective ability to address the climate crisis. Although we apply the same greenwashing definition, “. . .any communication that misleads people into adopting overly positive beliefs about an organization’s environmental performance, practices, or products,” as in Lyon and Montgomery (2015, p. 226), our scope is very different from that of the previous review. We review work that has emerged since 2014 (the last year in the prior review) through August 2022, explore why greenwash has not abated, highlight new forms of greenwash that were unanticipated by our review, and offer a more cohesive theoretical model of greenwashing.

To achieve our objective of motivating future research to understand and tackle new and emerging forms of greenwash, after discussing our methodology for the current review, we go on to discuss the literature as developing through three key phases. We see these as being somewhat temporal in nature, but also illustrating the growing theoretical development of the literature as well as the shifting forms and mechanisms of greenwashing. We begin briefly with what we term *Greenwashing 1.0: Static Communication to Consumers*. We see this phase as what we sought to summarize in 2015, capturing research and real-world approaches that largely focused around consumer-facing greenwashing, often through advertising or packaging, with a relatively straightforward set of techniques that have been captured and explained well in the literature and reviews. However, there have been some new developments in assessing the profitability and the environmental impacts of this form of greenwashing, which we discuss.

We next move to *Greenwashing 2.0: Dynamic Management of Stakeholders, Issues, and Intermediaries*. We see this second and current phase in the literature, and in business practice, as one where greenwashing has become broader, new techniques and mechanisms have been added, and new stakeholders or intermediaries have become involved and/or been affected by greenwashing. Here, we do not seek to be exhaustive, as other current reviews have also discussed this phase, but rather to use the benefits of our long-term view of the literature to highlight three major theoretical departures from Greenwashing 1.0: (a) Managing Stakeholders, including the impact of stakeholder pressures on greenwashing behavior, attention to the role of cognition, and the growth of brownwashing; (b) Managing Intermediaries, in which we focus on the changing role of certifications and their potential to facilitate greenwash; and (c) Managing New Issues, which highlights the effects of greenwash in the political domain and the emergence of “wokewashing” and other new variants. The areas we highlight are ones that research has begun to explore but that are far from exhausted, leaving many openings for impactful work.

Last but certainly not least, we seek to move beyond the parameters of a typical backward-looking review as we tackle *Greenwashing 3.0: Creation of Narratives about the Future*. In this section, we rely less on our review of published and peer-reviewed papers. Instead, we seek to draw together insights from working papers in progress that are being presented at leading conferences and related papers that fall beyond the boundaries of our structured review. In addition to academic work, we also scan and incorporate important developments in media and practitioner outlets on greenwashing. Here, although we seek to avoid terminology proliferation, we feel obligated to call attention to “futurewashing” as a new approach, and one to which researchers, stakeholders, and policymakers should be particularly attuned. Net zero commitments are by nature long-term promises, rather than verifiable statements about current performance, and open up a whole new world of greenwashing mechanisms.

Finally, we seek to bring together insights the literature has gained during these three phases to begin to develop a dynamic model of misleading environmental communications. Here, we incorporate emerging insights on how firms manage to (mis)communicate to different stakeholders and which stakeholders may be most likely to be deceived or greenwashed. By their nature, our 3.0 section and our proposed model are more speculative in nature. As scholars who care deeply about the real-world impacts of greenwashing, and see a vital role for cutting-edge and rigorous research to help to tackle and expose greenwash, we believe these efforts are important as the profession pushes this research into the future and continues to create real-world change and impact on greenwash practices.

## Method

To understand how the discourse on greenwashing has developed since our last review, in the first phase of our literature review we conducted quantitative searches across Web of Science (academic journals), Factiva (popular press), EBSCO (academic and trade journals) and ABI/Inform (academic and trade journals) on the term “greenwash.” These searches showed some fascinating general trends in the interest in, and publications on, greenwashing in academic, trade, practitioner, and news outlets. We used a variety of databases due to the different foci and specialties of each, and we display results from these four key databases in Figure 1. Our full review methodology is captured in Table 1.

**Table 1.** Review Methodology.

Phase	Focus	Description
Phase 1:	Exploratory analysis of trends	The first phase of our search retraces changes to the literature since our last review in 2015
	*Step 1*: Quantitative development	We carry out a *full-text* search^a^ across EBSCO’s Business Source Complete and the ABI/Inform Global database for articles containing “greenwash” or alternative forms/spellings (see Illustration X). —Scholarly articles (after removing duplicates): 2,890
	*Step 2*: Literature reviews etc.	We iteratively identify^a^ (a) reviews, and articles that otherwise (b) synthesize or (c) expand the existing greenwashing space. —Number of review articles: 12
Phase 2:	Demarcation of greenwashing	The second phase of our search captures the breadth of the greenwashing literature
	*Step 3*: Exploratory search	We conduct a *full-text* search^a^ for “greenwash” and its alternative forms/spellings using EBSCO Business Source Complete, download pdfs, and extract paragraphs with the keywords. —Article count: 1,175
	*Step 4*: Initial keyword list	Within the extracted paragraphs, we search for all terms that could describe a form of greenwashing. These terms constitute our list of initial keywords.
Phase 3:	Selection of corpus	The third phase of our search begins to narrow down the literature into a corpus for deeper analysis
	*Step 5*: Iterative search	We use our initial keyword list to search Web of Science for articles^a^ that mention a form of greenwashing in the *titles, abstracts, and author keywords*. We limit this search to journals in the Social Sciences Citation Index and iteratively adjust the search to remove obvious false positives. —Article count: 657
	*Step 6*: Journal selection	To reduce the size of the corpus while retaining as many relevant articles as possible, we only keep articles that were published in journals ranked 3 or higher in the Chartered Association of Business Schools’ 2021 Academic Journal Guide (ABS List) as well as the 10% most cited journal articles by year. —Article count: 249
	*Step 7*: Manual review	We manually select relevant articles from the corpus for our review. —Article count: 182
Phase 4:	Quantitative analysis	The fourth phase of our research uses various techniques for analyzing and organizing the extant literature in our corpus.
	*Step 8*: Manual review	We iteratively code articles and then used these abstract codes to identify emerging themes.
	*Step 9*: Citation network analysis	We conduct a citation network of articles in our review via Litmaps (see Figure 2).
	*Step 10*: Explorative topic model	We create a topic model to illuminate qualitative changes to the literature using full journal articles that mention greenwashing, identified in EBSCO search in Step 1 (see Figure 3).

a In English-language journals, published between 2015 and August 2022.

To briefly summarize these findings, by 2018 the number of articles on greenwash published in the popular press (Figure 1, bottom right) and trade journals (Figure 1, bottom left) had dropped precipitously from the peak years of 2008 to 2009. Meanwhile, interest from scholars in academic journals (Figure 1, top row) appeared to be plateauing. However, in the years 2019 and after, the charts show an unexpected resurgence in interest in greenwashing articles across all publication types. In 2021, the number of greenwashing articles reached new record highs in all four panels of Figure 1. These data suggest that the conclusion of our 2015 review, with its hailing of an end to greenwash, was only partially correct. It is plausible that a backlash against greenwashing put pressure on companies that brought some forms of greenwashing to an end, reducing interest in the topic. The more recent increase, however, indicates a new development. It may be that firms have responded to the backlash by developing new forms of greenwashing, which in turn have incited new interest across audiences.

It has already been established that greenwashing encompasses a range of misleading communications that promote unduly positive beliefs regarding an organization’s environmental performance (Lyon & Montgomery, 2015). To capture this broad range, in Phase 2 of our review, we compiled a comprehensive list of search terms to be used in our final search. That two-step approach follows other reviews of fragmented fields (Marescaux et al., 2021; Oliveira & Lumineau, 2019). First, we used EBSCO’s Business Source Complete database to identify articles published in 2015 or after, through to August 2022, that mention “greenwash” or its alternative forms and spellings anywhere in the text. That first full-text search yielded 1,175 articles on a wide range of topics that mention but do not necessarily focus on greenwashing. We extracted from these articles the paragraphs that mention greenwashing. Based on these paragraphs, we compiled a list of terms that could describe a greenwashing activity. This list yielded new keywords such as “decoupling,” “deceptive labeling,” “cheating,” or “obfuscation.”

We then used our list of keywords to search titles, abstracts, and author keywords via Web of Science. We limited this search to articles published in 2015 or after (after our prior review) in journals included in the Social Sciences Citation Index, which covers over 3,400 journals in 58 different social science disciplines. This second search yielded a more concentrated list of 647 articles on greenwashing. From this corpus, we retained the 10% most frequently cited articles each year, as well as articles that were published in journals ranked 3 or higher in the Chartered Association of Business Schools’ 2021 Academic Journal Guide (ABS List).^1^ After manually excluding 26 articles that were clearly irrelevant (e.g., they were only tangentially related) from that subset, we were left with 156 articles in which a form of greenwashing played a prominent role. In a next step, and because even 156 may be overwhelming for many readers, we developed a condensed list of articles (see Table 2). Table 2 includes papers among the top 5% of articles that were most cited each year, ABS List 4* journals, and top articles from the present journal.

**Table 2.** Most Cited Greenwashing Articles, and Articles in Major Journals, 2015–2022.

Article	Title	Journal
Boiral et al. (2022) ^c^	Through the smokescreen of the Dieselgate Disclosure: Neutralizing the impacts of a major sustainability scandal	Organization & Environment
Carlos & Lewis (2018) ^a,b^	Strategic silence: Withholding certification status as a hypocrisy avoidance tactic	Administrative Science Quarterly
Comyns et al. (2022) ^c^	Cut them loose? Firms’ response strategies to environmental misconduct by supplying firms	Organization & Environment
Crilly et al. (2016) ^a^	The grammar of decoupling: A cognitive-linguistic perspective on firms’ sustainability claims and stakeholders’ interpretation	Academy of Management Journal
D’Arcy et al. (2020) ^a^	Too good to be true: Firm social performance and the risk of data breach	Information Systems Research
Fabrizio & Kim (2019) ^a^	Reluctant disclosure and transparency: Evidence from environmental disclosures	Organization Science
Fiechter et al. (2022) ^a^	Real effects of a widespread CSR reporting mandate: Evidence from the European Union’s CSR directive	Journal of Accounting Research
Halme et al. (2020) ^b^	When is there a sustainability case for CSR? Pathways to environmental and social performance improvements	Business & Society
Harjanne & Korhonen (2019) ^b^	Abandoning the concept of renewable energy	Energy Policy
Kassinis & Panayiotou (2018) ^c^	Visuality as greenwashing: The case of BP and Deepwater Horizon	Organization & Environment
S. Kim & Yoon (2023) ^a^	Analyzing active fund managers’ commitment to ESG: Evidence from the United Nations principles for responsible investment	Management Science
J. Kim et al. (2017)	Attention, action, and greenwash in Family-influenced firms? Evidence from polluting industries	Organization & Environment
E.-H. Kim & Lyon (2015) ^a,b^	Greenwash vs. brownwash: Exaggeration and undue modesty in corporate sustainability disclosure	Organization Science
M. Li et al. (2022) ^b^	The clean energy claims of BP, Chevron, ExxonMobil and Shell: A mismatch between discourse, actions, and investments	PLOS ONE
W. Luo et al. (2019) ^b^	Environmental information disclosure quality, media attention, and debt financing costs: Evidence from Chinese heavy polluting listed companies	Journal of Cleaner Production
L. Luo (2019) ^b^	The influence of institutional contexts on the relationship between voluntary carbon disclosure and carbon emission performance	Accounting & Finance
Lyon & Montgomery (2015) ^b,c^	The means and end of greenwash	Organization & Environment
Marquis et al. (2016) ^a,b^	Scrutiny, norms, and selective disclosure: A global study of greenwashing	Organization Science
Morales-Raya et al. (2019) ^c^	To be or to seem: The role of environmental practices in corporate environmental reputation	Organization & Environment
Momsen & Ohndorf (2022) ^b^	Information avoidance, selective exposure, and fake (?) news: Theory and experimental evidence on green consumption	Journal of Economic Psychology
Nardi (2022) ^a^	The corporate social responsibility price premium as an enabler of substantive CSR	Academy of Management Review
Rausch & Kopplin (2021) ^b^	Bridge the gap: Consumers’ purchase intention and behavior regarding sustainable clothing	Journal of Cleaner Production
Siano et al. (2017) ^b^	“More than words”: Expanding the taxonomy of greenwashing after the Volkswagen scandal	Journal of Business Research
Sterbenk et al. (2022) ^b^	Is Femvertising the new greenwashing? Examining corporate commitment to gender equality	Journal of Business Ethics
Supran & Oreskes (2017) ^b^	Assessing ExxonMobil’s climate change communications (1977–2014)	Environmental Research Letters
Szabo & Webster (2021) ^b^	Perceived greenwashing: The effects of green marketing on environmental and product perceptions	Journal of Business Ethics
Testa, Boiral, & Iraldo (2018) ^b^	Internalization of environmental practices and institutional complexity: Can stakeholders sressures encourage greenwashing?	Journal of Business Ethics
Torelli et al. (2020) ^b^	Greenwashing and environmental communication: Effects on stakeholders’ perceptions	Business Strategy and the Environment
van der Waal & Thijssens (2020) ^b^	Corporate involvement in Sustainable development goals: Exploring the territory	Journal of Cleaner Production
R. Wang, Wijen, et al. (2018) ^a,b^	Government’s green grip: Multifaceted state influence on corporate environmental actions in China	Strategic Management Journal
Z. Wang & Sarkis (2017) ^b^	Corporate social responsibility governance, outcomes, and financial performance	Journal of Cleaner Production
Wu et al. (2022) ^b^	Substantial response or impression management? Compliance strategies for sustainable development responsibility in family firms	Technological Forecasting and Social Change
Wu et al. (2021) ^b^	Generous charity to preserve green image? Exploring linkage between strategic donations and environmental misconduct	Journal of Business Research
Wu et al. (2020) ^a^	Bad greenwashing, good greenwashing: Corporate social responsibility and information transparency	Management Science
Xing et al. (2021) ^b^	Green credit policy and corporate access to bank loans in China: The role of environmental disclosure and green innovation	International Review of Financial Analysis
Yu et al. (2020) ^b^	Greenwashing in environmental, social and governance disclosures	Research in International Business and Finance
Zhang (2022a) ^b^	Are firms motivated to greenwash by financial constraints? Evidence from global firms’ data	Journal of International Financial Management & Accounting
Zhang et al. (2018) ^b^	The influence of greenwashing perception on green purchasing intentions: The mediating role of green word-of-mouth and moderating role of green concern	Journal of Cleaner Production

*Note.* ESG = environmental, social, and governance; ABS = Association of Business Schools; CSR: Corporate Social Responsibility.

a Articles in ABS 4* journals. ^b^Top 5% most cited articles (by year). ^c^Most cited O&E articles (by year).

**Figure 2.**
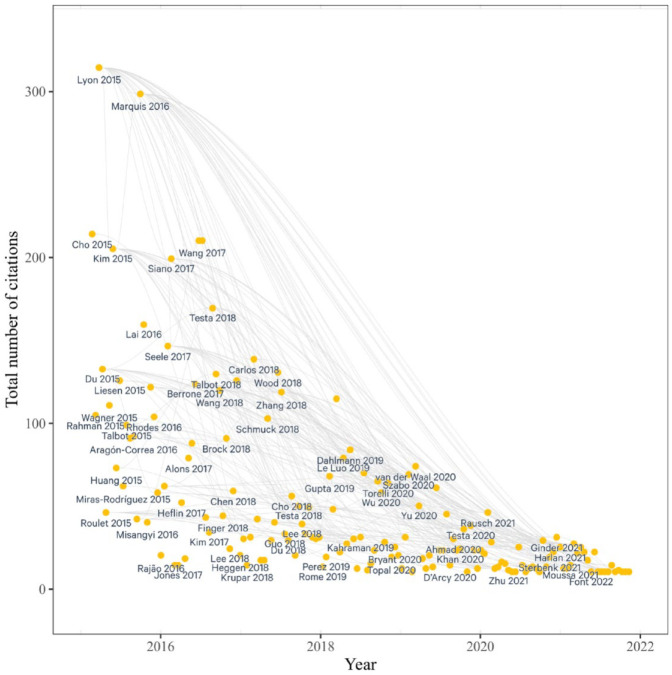
Citation Network Analysis (Papers Published Post-2014). *Note.* Each line shows an instance where one article in our sample is cited by another article in our second search. Litmaps uses the Crossref and Semantic Scholars APIs to determine both where an article is cited, and what other article it cites. The x-axis shows how often the article was cited by other articles inside or outside of the sample. An article with a denser number of connections is more central to the greenwashing literature, while an article with a high total number of total citations but few network connections may be tangential to greenwashing and part of a different conversation. For instance, Wang and Sarkis (2017) are well-cited, but not very central to the greenwashing literature as captured by our search. Meanwhile, Testa, Boiral and Iraldo (2018) are more central: they cite articles that came before in our sample and are cited by works that came after.

To further understand the nature of the literature and how it had evolved in recent years, we also conducted a citation network analysis which illustrated connections across papers as well as new lines of literature (see Figure 2). We imported the list of articles from our second search into Litmaps and identified articles that are central to the citation network, that is, articles that cite many preceding greenwashing works and are cited by subsequent works. We then used the citation network analysis, essentially the wisdom of the field, to test our own intuition on past and emerging trends in the literature, and to inform the next step in our analysis.

In the third phase of our analysis, we manually coded our sample of 156 articles to highlight qualitative developments in the literature, seeking to identify emerging themes. We coded the papers iteratively and ended the process when we reached saturation, that is, no new constellations emerged and we were not able to integrate more articles into the existing groups. Table 3 shows all papers we were able to organize in this fashion and key themes that emerged, along with the actor committing greenwashing, the actor targeted by greenwashing, and the medium of greenwashing. We then used the codes to organize the sample into three overarching phases which informed the structure of our thinking and of the paper itself: Greenwashing 1.0, 2.0, and 3.0 as outlined in Table 3 and in the text.

**Table 3.** Actors, Targets, and Mediums in Greenwashing 1.0, 2.0, 3.0.

Greenwasher	Target	Medium	Description	Examples
Greenwashing 1.0				
Firm	Consumers	Marketing	Misleading marketing and its effect on consumers.	Szabo & Webster (2021); Schmuck et al. (2018); Y.-S. Chen, Huang, et al. (2020); Rausch & Kopplin (2021)
Firm	Customers	Products, services, and packaging	Products, services, and packaging can be designed to mislead customers.	Momsen & Ohndorf (2022); Testa et al. (2020); Gupta et al. (2019); Kahraman & Kazançoğlu (2019); Siano et al. (2017); Rahman et al. (2015)
Firm	Consumers	Certification	Can consumers tell if eco-certifications is credible or greenwashing?	Gosselt et al. (2019)
Firm	Stakeholders and public	ESG communication	Firms commit to environmental practices and publish environmental information to attain a good image.	Morales-Raya et al. (2019); Coen et al. (2022); Torelli et al. (2020); Ruiz-Blanco et al. (2022)
Firm	Banks, investors, and financial markets	ESG communication	Firms benefit from disclosing ESG data or adopting symbolic practices without making substantive changes.	L. Luo (2019); Wellalage & Kumar (2021); Xing et al. (2021); H.-Z. Khan et al. (2023); W. Luo et al. (2019); Sterbenk et al. (2022; see also Z. Wang & Sarkis, 2017)
Firm	Government and stakeholders	CSR	Firms engage in environmental projects to gloss over negative impacts related to their core business.	Wu et al. (2020); Halme et al. (2020); Leung & Snell (2017)
Firm	Public	Charity	In response to an environmental issue such as a pollution incident, firms may donate to charity to improve their image.	Wu et al. (2021); Wu et al. (2022)
Firm	Various	Emission disclosure	Firms disclose emissions data to gain legitimacy; government, NGOs, and the media push back.	Marquis et al. (2016); Du (2015); W. Luo et al. (2019); L. Luo (2019); Yu et al. (2020); R. Huang & Chen (2015); Fiechter et al. (2022; see also Nardi, 2022)
Firm	Stakeholders	ESG disclosure	Which firms are more likely to greenwash?	Terlaak et al. (2018); Marquis et al. (2016); L. Luo et al. (2018; see also D’Arcy et al., 2020)
Greenwashing 2.0				
Firm	Stakeholders	Environmental action and communication	Firms may consider environmental action and communication independent and deliberately undercommunicate/brownwash to avoid perceived incongruence.	E.-H. Kim & Lyon (2015); Ginder et al. (2021); Y. Huang et al. (2022); Testa, Miroshnychenko, et al. (2018)
Firm	Shareholders	Certifications and ESG commitments	Shareholder pressure determines how companies engage with certifications and past commitments–whether they publish their certification status and internalize practices.	Carlos & Lewis (2018); van Halderen et al. (2016); Testa, Boiral, & Iraldo (2018); J. Kim et al. (2017)
Firm	Government	CSR report	When firms are required by the government to publish CSR reports, they find inexpensive ways to comply, and create positive data.	Leung & Snell (2017); H.-Z. Khan et al. (2020); Leung & Snell (2021)
Firm	Stakeholders	ESG information disclosure	When companies are under financial distress, their ESG disclosure is more likely to be a greenwashing effort and less likely to lead to emission reduction.	Zhang (2022a); Zhang (2022b)
Firm	Stakeholders	Website	After an environmental disaster, BP reacts to criticism by avoiding specific statements that could be scrutinized but instead relies on imagery presenting itself as scientific, professional, and responsible.	Kassinis & Panayiotou (2018)
Government	Firms or public	Policy	Other policy objectives interfere with green policy, leading to greenwashing.	R. Wang, Wijen, et al. (2018); Harlan (2021); Harjanne & Korhonen (2019); Hufen (2016)
Mutual funds	Investors	ESG funds	Signees of the UN Principles for Responsible Investment attract more funds but do not improve their holdings’ ESG performance.	S. Kim & Yoon (2023)
Firms	Media and public	ESG investing	By not doing their due diligence, journalists become complicit in greenwashing.	Strauß (2022)
Government	Public	Marketing	The government paints its effort to curb industrial pollution in a positive light.	Alon-Barkat (2020)
Community and government	Firms and consumers	Carbon market	Actors can shape the rules of emission abatement to attain financial benefits.	Rajão & Marcolino (2016); Hufen (2016)
Firm	Stakeholders and public	Certification	Certifications were created in response to environmental issues, but companies can become certified without going green.	Liute & De Giacomo (2022); Flowers et al. (2020); Garrido et al. (2020); Heras-Saizarbitoria et al. (2020); Wagner (2022)
Firm and government and/or community	NGOs and activists	Various	When an organization provides resources to other actors in its community, these actors are likely to support the organization and come to its aid.	Crilly et al. (2016); Brock & Dunlap (2018)
Greenwashing 3.0				
Firm	Politicians and the public	Lobbying	Firms publicly support environmental protection but lobby against relevant environmental policy.	Lyon et al. (2018); Cho et al. (2018)
Firm	Stakeholders	Certified ESG disclosure	Within highly scrutinized disclosure schemes, firms do not use positive information to greenwash, but obfuscate their negative impacts, e.g., through complexity.	Fabrizio & Kim (2019); Talbot & Boiral (2018); Z. Wang, Hsieh, & Sarkis (2018); Shvarts et al. (2018)
Firm	Government and public	Pledges and goals	Firms pledge to reduce their environmental impacts, which may buy them time to continue polluting before promises are weighted against progress.	M. Li et al. (2022); van Halderen et al. (2016); Dahlmann et al. (2019); van der Waal & Thijssens (2020)
Firm	Stakeholders	Environmental misconduct	Stakeholders can only observe whether prior statements were grandstanding or genuine when a firm is confronted with its involvement in an unethical industry practice and pressed to make changes.	Comyns et al. (2022); Boiral et al. (2022)
Firm	Public and government	Pilot projects and future technologies	New technology for emission abatement make government interventions appear unnecessary, but companies may not follow through beyond pilot plants.	Lebelhuber & Greiling (2022); M. Li et al. (2022); Brock & Dunlap (2018)
Firm	Public	Media	Oil companies enter the public discourse to downplay or deny the climate impacts of fossil fuel.	Supran & Oreskes (2017); van Halderen et al. (2016)
Sustainability managers	Stakeholders	Stakeholder communication	Sustainability managers may not be clear about their environmental impacts and make sweeping, unspecific claims. Only specialist stakeholders can see through the claims.	Crilly et al. (2016); Talbot & Boiral (2015)

*Note.* ESG = environmental, social, and governance; CSR = corporate social responsibility; NGO = non-governmental organization.

**Figure 3.**
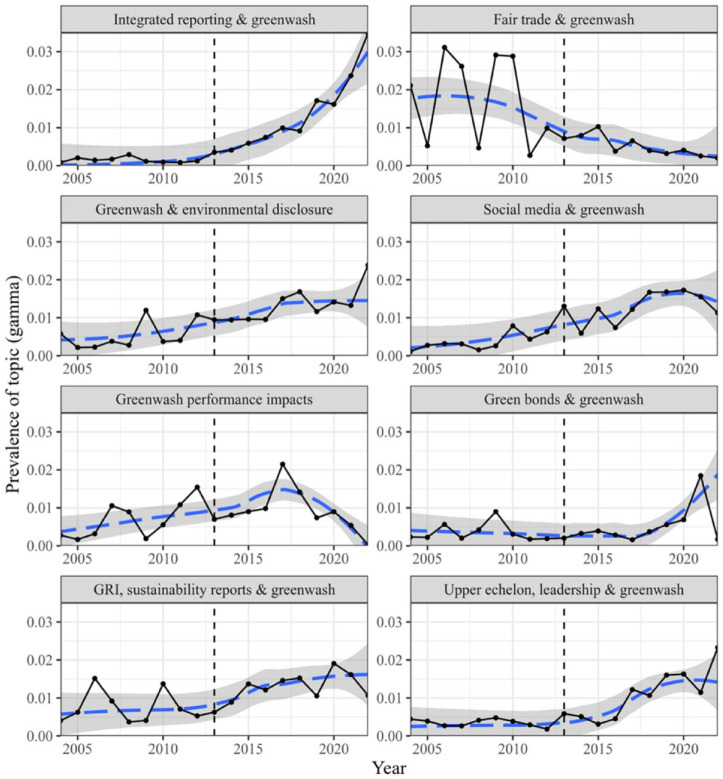
Exploration of Qualitative Changes to the Literature Through Topic Modeling. *Note.* Solid black line: mean of all articles in 1 year. Dashed blue line: local regression trend line with confidence interval. The dotted vertical line marks the status of the discourse at the time of our last review (Lyon & Montgomery, 2015).

Although Greenwashing 1.0 and Greenwashing 2.0 rely on extant literature and were analyzed by systematically identifying key themes characterizing each phase. In contrast, Greenwashing 3.0 features misleading communications that we see as newer and still emergent, such as firms using uncertainty about the future as a vehicle for greenwashing by pledging future action that may never arrive. Although we have identified some literature (mostly non-academic) that describes these activities, we expect these activities to become much more prominent as anthropogenic climate change progresses. As this 3.0 section draws much less on our review of extant published papers, here we employed a final empirical review technique to draw out and support our analysis on these emerging trends. We created a topic model from 1,847 full journal articles mentioning greenwashing from the EBSCO Business Source Complete database. We used the topic model to conduct a historical analysis and identify trends in the literature across time (Hannigan et al., 2019). Figure 3 shows a subset of these trends, which characterize the qualitative development of the literature since our last review. These trends indicate that the greenwashing literature has not only grown in magnitude but also evolved in terms of the phenomena and topics discussed. Perhaps most strikingly, articles on the role of greenwash in integrated reporting have grown very rapidly in recent years, while articles on greenwash and Fair Trade have fallen off. In sum, the current discourse focuses less on specific products, industries, and certifications—such as Fair Trade or green building products—than it did at the time of our last review. Instead, the focus is shifting to greenwashing at a grander scale, in particular through ESG and related phenomena, which in part inspired the Greenwashing 3.0 section of this article.

## Greenwashing 1.0: Static Communication to Consumers

Research in this initial phase treated greenwashing predominantly as a unidirectional process aimed at consumers, and had two broad themes. The first theme was the elaboration of the classic greenwashing model of a firm misleading an audience and the mechanisms involved. The second theme was the identification of variables that can predict greenwashing, or shine a spotlight on actors that can curtail greenwashing; in line with the theme of our prior review, these papers look for the “end of greenwashing.”

Several other important reviews have appeared since ours, each of which also contributed to the development of theory and to aligning and deepening the scholarly understanding of greenwashing. We see most of this work, like that covered by our earlier review, as focusing on what we term Greenwashing 1.0—largely product-level and consumer-focused greenwashing, with the gradual introduction of new stakeholders and new means of greenwashing. For example, Gatti et al. (2019) provide a broad and thorough review, summarizing past literature and proposing the use of voluntary and mandatory measures to address greenwashing. Another review, by de Freitas Netto et al. (2020), develops a typology of different forms of greenwashing and definitions that emerged in the prior 10 years, offering a helpful summary of the literature. An interdisciplinary review by Nemes et al. (2022), whose authors include natural and social scientists and practitioners, sought to integrate past literature with the aim of developing a framework for assessing greenwashing that can be used by practitioners to avoid or call out such claims. In short, a growing and increasingly rigorous body of research has teased out, tested, and categorized the “means” of greenwashing relatively thoroughly, and this work has also been well summarized and reviewed in prior work we needn’t repeat herein. As a result, it has become increasingly difficult to make substantive contributions to this part of the literature. Even such time-tested strategies as inventing a new catch-phrase to describe a familiar phenomenon are unlikely to go far as future research endeavors.

*Despite these caveats, there are two themes within the Greenwashing 1.0 literature that have emerged since our prior review and that merit further attention. Both themes call into question certain assumptions that have been largely taken for granted in the literature. The first questions whether greenwash is, in fact, profitable for the firms that engage in it. The second questions whether greenwash is, in fact, harmful for society.*

### Is Greenwash Profitable?

It is natural to assume that greenwashing must be profitable or firms would not do it. However, it is conceivable that greenwash is profitable in the short term but not in the long term, and that it represents an agency problem, with marketers engaging in greenwash in an attempt to advance their own careers at the expense of shareholders. The empirical literature on the profitability of greenwash is small and the results it reports are not entirely consistent, suggesting that context matters greatly. Research using Chinese data suggests that greenwash is profitable (W. Li et al., 2023), but research using a broader sample of firms from 58 different countries suggests that greenwash has no significant effect on profits while brownwash has a negative and significant effect on profits (Testa, Miroshnychenko, et al., 2018). Other work using firms from a broad sample of countries suggests that the financial impacts of greenwash depend upon the stakeholder it targets, with no impact when targeted to internal stakeholders but a positive financial impact when targeted to external stakeholders (Schons & Steinmeier, 2016). Finally, there is some evidence that the maturity of firm matters. Early-stage startups in the European Union showed no financial impacts from either greenwash or brownwash, but firms in existence for 8 to 10 years suffered a negative profit impact from greenwash, although brownwash continued to have no effect. (Neumann, 2021). Further work to clarify the mechanisms at play would be worthwhile.

### Is Greenwash Always Bad?

Although there are clear reasons to be concerned about the negative impacts of greenwash, recent research suggests that under certain conditions greenwash may actually have positive effects. Greenwash can be good for consumers, even though it blurs quality differences between products, if it sufficiently intensifies price competition between firms with market power (Piccolo et al., 2015). Greenwashing of labels can be good for the environment if it intensifies price competition between labeled products and induces some consumers to switch from completely brown products to greener labeled products (Heyes et al., 2020). Greenwashing can also be good for the environment if it largely reaches “brown” consumers who seldom choose with the environment in mind and motivates them to purchase greener products than they would otherwise, instead of reaching “green” consumers and misleading them into purchasing browner products (Chan, 2015). Moreover, the ability to greenwash can encourage brown firms to increase their green investments (Glaeser & Ujhelyi, 2010), although it can also deter green firms from increasing theirs (Wu et al., 2020). Finally, brown firms may initially engage in greenwashing, but if norms around transparency later tighten up those firms may be pressured to upgrade their performance (Haack et al., 2021), leading to a “recoupling” of claims and behavior.

## Greenwashing 2.0: Dynamic Management of Stakeholders, Intermediaries, and Issues

As the literature on Greenwashing 1.0 began to establish a coherent framework of activities and mechanisms, the goal posts shifted. Although this is in part due to the literature maturing and researchers asking new and different questions, it is also at least in part due to growing interest in sustainability—or being “green”—on the part of stakeholders beyond the traditional target audience of consumers. With pressures increasing from employees, investors, government, social movements and others for firms to step up their green credentials and reporting, greenwashing likewise shifted. Research has kept pace with these shifts, and in this section, we identify several key trends and patterns in the literature. Again, we do not seek to be exhaustive, but we focus on three key themes we identified in this emergent research: (a) *Managing Stakeholders*, including the impact of stakeholder pressures on greenwashing behavior, attention to the role of cognition, and the growth of brownwashing; (b) *Managing Intermediaries*, in which we focus on the changing role of certifications and their potential to facilitate greenwash; and, (c) *Managing New Issues*, which highlights the effects of greenwash in the political domain and the emergence of “wokewashing” and other new variants. Importantly, we also note that none of these topics has yet been exhausted or fully developed and suggest potential avenues for future research in order to continue to develop and explore these important and promising topics.

### Managing Stakeholders

Greenwashing 2.0 has tended to focus on dynamic and interactive relationships, meaning that greenwash is no longer a one-way process occurring in a vacuum. Rather, firms constantly interact with their environment, and respond to demands and critiques from stakeholders. In this section, we begin with an introduction on multiple stakeholders and then expand to emerging issues including brownwashing, stakeholder cognition, and emerging denial and doubt strategies.

#### Multiple Stakeholders

**Figure 4.**
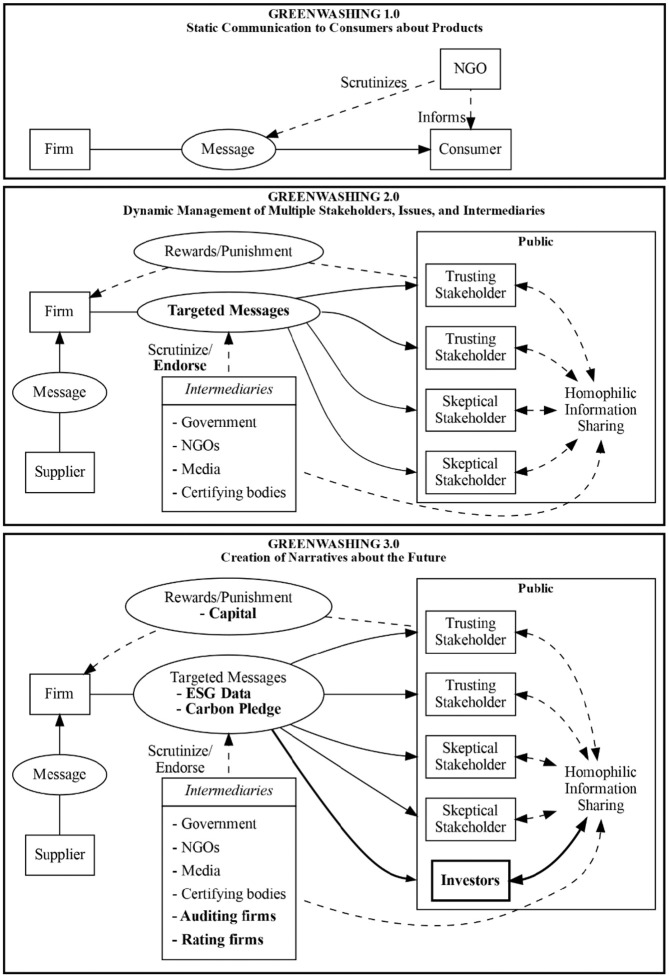
A Proposed Model of Corporate Miscommunication. *Note.* Bold font highlights significant changes between greenwashing 1.0, 2.0, and 3.0.

A main focus of the recent greenwashing literature is the role of previously overlooked actors in the greenwashing process (see Figure 4). The majority of the papers we reviewed include at least one actor outside the greenwashing 1.0 triad of firms, consumers, and NGOs (see Table 3). These additions challenge the previous conceptualization of greenwashing as relatively static. Greenwashers can for instance apply multiple strategies in parallel, and greenwashing naturally takes a different form depending on the actors involved. The most salient additions to the literature are discussions on the role of governments (e.g., Alon-Barkat, 2020; R. Wang, Wijen, et al., 2018) and supply chains (e.g., Comyns et al., 2022; Pizzetti et al., 2021). One emerging focus of this growing line of work on greenwashing and multiple stakeholders is the differing demands of often competing stakeholders (e.g., Torelli et al., 2020), and how firms pursue their own objectives in such a fraught landscape.

A dynamic view on greenwashing has far-reaching implications, as best illustrated by new research on the role of governments. The different functions government plays, for example, illustrate the difference between a static and a dynamic view of stakeholders and greenwashing in the literature. In a static model, the government would primarily act as a constraint on greenwashing behavior that targets another audience (e.g., L. Luo, 2019; Marquis et al., 2016; Shanor & Light, 2022; Yu et al., 2020). In a dynamic model, the government acts both as a constraint and as an impetus for novel greenwashing behavior by firms. For instance, government initiatives around CSR, such as India’s requirement that firms spend 2% of their profits on corporate social responsibility (CSR), may prompt firms to develop new rhetoric on environmental sustainability (Prasad, 2014). That rhetoric may subsequently act as a smokescreen, preventing other stakeholders from understanding those firms’ actual environmental impacts (H.-Z. Khan et al., 2020; Leung & Snell, 2017, 2021).

There are also more radical departures from the original firm-consumer greenwashing model, showing that non-firm actors may also have an interest in misleading people about environmental outcomes. Alon-Barkat (2020) studies a large-scale advertising campaign for a government’s air pollution abatement policy which failed to reduce air pollution or improve air quality. Governments often derive their legitimacy from economic growth for their constituency and may also choose to turn a blind eye when firms violate environmental policy (J. Wang, Jiang, et al., 2021). Moreover, other stakeholders may side with greenwashers if they have shared interests. This process is most salient when firms provide resources to stakeholders (Brock & Dunlap, 2018; Crilly et al., 2016). As a result of a shared interest around corporate sustainability, the dividing line between greenwashers and their stakeholders has become a gray area. For example, financial journalists have covered extensively the rise of ESG investing and ESG goals and could be considered greenwashing accomplices based on their enthusiastic and often uncritical coverage of the sustainable finance trend (Strauß, 2022). Many U.S. mutual funds are incorporating ESG data into their investment decision-making processes. These funds successfully attract funding, but thus far their engagement with firms on ESG issues has not resulted in significant ESG performance improvements (S. Kim & Yoon, 2023).

In global supply chains, the dividing line between greenwasher and accomplice has long disappeared since firm–supplier relationships make it very difficult for other stakeholders to identify responsible actors and attribute blame for environmental misconduct (Comyns et al., 2022; Pizzetti et al., 2021). Perhaps the most extreme example of the new gray areas is the use of carbon credits intended to fund the removal of carbon dioxide from the atmosphere. For example, research has examined how corporate threats to deforest have been used to claim carbon credits simply by maintaining the status quo, a cynical ploy to get around requirements that offsets be “additional” to existing efforts (Hufen, 2016; Rajão & Marcolino, 2016).

#### The Varieties of Stakeholder Cognition

As mentioned above, the recent literature explores how greenwashing and brownwashing behavior is affected by the salience of various stakeholder groups. E.-H. Kim and Lyon (2015) find that firms are more likely to greenwash when they are growing and hence may need regulatory approval for new plants, and more likely to brownwash in deregulated markets, especially when their financial performance is poor; either way, distortions in disclosure are smaller when the firm is exposed to more scrutiny from environmental activists. Carlos and Lewis (2018) find that firms are less likely to publicize their membership in the Dow Jones Sustainability Index when they have recently been the subject of environmental shareholder resolutions or criticism from environmental activists, suggesting that pressure from investors and activists can prompt a firm to brownwash. Testa, Miroshnychenko, et al. (2018, p. 287) study whether firms engage in meaningful implementation of environmental management systems, and find that “while pressure from suppliers and shareholders contributes to corporate greening, pressure from customers and industrial associations tends to encourage greenwashing.”

The growing awareness that stakeholder salience shapes greenwashing behavior has prompted researchers to delve deeper into the cognitive characteristics that make particular stakeholder groups vulnerable to particular forms of greenwash. One particularly important issue is understanding when stakeholders will expend effort to verify the claims made by firms and the meaning of certifications firms obtain. Economic theory based on rational actors suggests that verification efforts are most likely from stakeholders who place the highest valuation on the sustainability attributes of a claim, and that stakeholders with moderate valuations may be influenced by claims even though they are not willing to incur the costs of verification (Heyes et al., 2020). However, the literature on “symbolic management” has made it clear that even investors with large amounts of funds at stake often fail to undertake even rudimentary levels of due diligence, such as bothering to read the details on whether a firm actually issued its CEO stock options when it claimed to have done so (Westphal & Zajac, 1998). Thus, it is crucial to consider the cognitive limitations of stakeholders when considering greenwash.

Perhaps the most obvious cognitive limitation is the simple fact that attention is a scarce resource. Recent work in finance has studied the effects of “distracted” institutional investors, that is, investors who are experiencing a shock in one portion of their portfolio (Kempf et al., 2017). As these investors devote more attention to this portion, they pay less attention to other parts of the portfolio. One interesting implication is that CSR performance declines in these other parts of the portfolio when investors are distracted (T. Chen, Dong, & Lin, 2020). In a similar fashion, investor distraction should facilitate greenwashing, but we are not aware of research that has applied this perspective to date.

One cognitive limitation that has attracted research attention is the inability to deal with linguistic complexity. Whether this makes complexity a tool for defeating or delivering greenwash, however, is currently a subject of debate. On one hand, Crilly et al. (2016) suggest that greenwashers use simple language. Drawing on a sample of 119 interviews with employees in 12 different companies, they suggest that firms delivering substantive achievements (“implementers”) use more complex language than firms that are merely providing lip service to their stated goals (“decouplers,” or “greenwashers” in our language). They also suggest that only certain kinds of stakeholders can distinguish between these two forms of discourse. Stakeholders who are specialists in the domain being discussed are better able to identify greenwash than are generalists, but stakeholders who have a conflict of interest that makes them want to believe certain optimistic claims are less likely to spot greenwash. On the other hand, Fabrizio and Kim (2019) suggest that greenwashers use more complex language to obfuscate. They build on a long tradition of work in computational linguistics to create a “fog index” capturing the complexity of language used by over 1,500 companies that voluntarily disclose climate information to the CDP. They find that firms with negative information to disclose use foggier language, and that the use of such language buffers the negative impact of bad news on the firm’s environmental ratings. Reconciling these opposing views on the impact of linguistic complexity is a worthy topic for further research.

#### Brownwashing, Obfuscation, and Silence

Research on greenwashing has done an excellent job of capturing the many pressures on firms that can lead to the temptation to greenwash. In light of these, it may seem paradoxical that many firms are, instead, downplaying or being modest about their sustainability efforts. Studying electric utilities and their reporting of greenhouse gas reductions, E.-H. Kim and Lyon (2015) coined the term “brownwashing” to capture under-reporting of environmental accomplishments. A series of related recent papers has further fleshed out the phenomenon of downplaying environmental achievements, proliferating new terms such as “strategic silence” (Carlos & Lewis, 2018), “greenhushing” (Ginder et al., 2021), or simply being “quiet” in the realm of philanthropy and CSR (H. Wang, Jia, & Zhang, 2021). Emerging research has now found that brownwashing has negative impacts on firms, including lower financial performance (Testa, Miroshnychenko, et al., 2018), and authors speculate it may also be detrimental to environmental progress more generally (Y. Huang et al., 2022). We, therefore, included brownwashing here as an important development in the broader greenwashing and environmental miscommunication literature.

Although still small, the brownwashing literature has begun to provide important insights and evidence on the drivers, circumstances and mechanisms of brownwashing. As with greenwashing, many of these are linked to stakeholder pressures, and the literature suggests brownwashers may be avoiding pressures from investors, customers, employees, peers, or the community (Delmas & Grant, 2014; Ferrón-Vílchez et al., 2021; Y. Huang et al., 2022; E.-H. Kim & Lyon, 2015; Koh et al., 2018; H. Wang, Jia, & Zhang, 2021). For example, firms may brownwash to avoid being labeled or categorized disadvantageously by stakeholders. In early work examining the role of employee and civil society pressures, Snellman (2012) found that Finnish firms downplay their adoption of a stronger shareholder value orientation when they face pressure from labor unions. Similarly, California wineries have downplayed their adoption of organic practices for fear of receiving a negative price premium due to the historic stigmatization of organic wine (Delmas & Grant, 2014). Stigma may have been a motivator of brownwashing in other consumer goods markets as well, due to concerns that environmentally friendly products may provide lower levels of performance.

Emerging research in this area has also begun to tease out the theoretical groundings of brownwashing to better capture the complexity of reactions to stakeholder pressures. Carlos and Lewis (2018) focus on firms that are “silent” about the fact that they have been ranked on the Dow Jones Sustainability Index, in what they find to be an effort to avoid accusations of hypocrisy. Similarly, Ginder et al. (2021) show that perceived incongruence between message and performance is seen as worse than bad performance by some audiences, and brownwash can alleviate the risk of consumers perceiving incongruence. H. Wang, Jia, and Zhang (2021) focus on what they term “quiet giving,” or philanthropic activities that are withheld from the scrutiny of a mixed array of stakeholders such as employees, investors, and the local community. Drawing on legitimacy and stakeholder theories as underlying motivations, those authors argue that firms strive to balance tensions across stakeholders. With similar objectives and a large empirical study, Y. Huang et al. (2022) find that firms brownwash to avoid peer pressure and excessive attention from stakeholders, and to maintain industry leadership positions. Montgomery and Robertson (2022) go beyond the general focus on negative reactions to stakeholder pressures and develop a model of both positive (“proactive”) and negative (“reactive”) responses, as well as reasons for brownwashing that may be operational and have little to do with stakeholders (“indifferent”).

In sum, the brownwashing discourse highlights that firms are part of a dynamic context including multiple stakeholders whose interests must be balanced against one another (E.-H. Kim & Lyon, 2015). In this dynamic context, a firm’s decision to tout its sustainability credentials becomes but one decision in an interdependent series of environmental management decisions and interactions with stakeholders (Carlos & Lewis, 2018; Montgomery & Robertson, 2022).

#### Blame Shifting, Denial, and Doubt

A literature has begun to emerge that examines a variety of corporate tactics to divert attention from corporate and industry “bad actors,” and raise questions about their responsibility for social outcomes. For example, Aronczyk and Espinoza (2022) examine how public relations firms employed by Big Oil have attempted to shift the blame for the climate crisis, while others have highlighted how these firms have pursued the deliberate shifting of responsibility for “carbon footprints” from corporations to individuals (Kaufman, 2020). These forms of greenwashing are by no means new, and were perhaps made most famous by the tobacco industry in a series of tactics captured in Oreskes and Conway’s (2010) aptly named book, *Merchants of Doubt*. Yet the study of denial and doubt as tools is relatively new to the greenwashing literature. As Talbot and Boiral (2015) summarize, companies may argue that they are not brown when compared with other firms that emit even more, that emission reductions would be infeasible, that stricter regulations would be economically irresponsible, or that climate change would not hurt their region. These companies are not claiming to be green. Rather, they raise doubt about what it means to be brown. In particular, on the issue of climate change, some companies have gone beyond doubt and dabbled in denial. Following the adoption of the 1997 Kyoto Protocol, an alliance of industry actors demonstrated the power of climate denial by defeating some of the first climate change legislation (Mann, 2012).

Actors can use a range of techniques to mislead audiences about climate change and sow doubt. Like greenwashing, denial may take the form of selective disclosure. One of the first documented instances of climate denial, in 1980, used out-of-context material from a study to suggest that human activities could lead to global cooling (Franta, 2021). Climate denial can also take the shape of decoupling or outright deception—Supran and Oreskes (2017) point out that ExxonMobil had until 2004 regularly paid for advertorials in the *New York Times* that questioned whether climate change is real, or whether it is caused by humans, even though internal documents indicate that the company had little doubt on the matter (Supran et al., 2023). Interestingly, Exxon (pre-merger) also greenwashed donations to climate skeptics by donating US$100 million to climate research at Stanford (van Halderen et al., 2016). Again, climate denial is not suitable for making a company appear green, but it can put into doubt whether fossil fuels are really brown and whether it is worthwhile to implement climate policy.

Brock and Dunlap (2018) highlight that corporations may present themselves as part of the solution rather than the problem, for instance by exhibiting carbon capture technology. By alluding to future climate action, a tactic we discuss in more detail in our Greenwashing 3.0 section, companies can enhance their image and simultaneously avert the impression that more regulations are necessary. Scholarship has woken up to the fact that heavy polluters like BP often adorn themselves with science-related imagery and green energy slogans (“we care about generations to come”) but have made few specific promises (Kassinis & Panayiotou, 2018). BP excited critics with its “Beyond Petroleum” rebranding, though without ever significantly investing in renewable energy (Ferns & Amaeshi, 2021; van Halderen et al., 2016). BP, Chevron, ExxonMobil, and Shell all frequently discuss low-carbon technologies in their annual reports, but their clean energy spending is insignificant and opaque (M. Li et al., 2022; Parafiniuk & Smith, 2019). Pilot projects in particular blur the line between rhetoric and action. Pilot projects exhibit real technology, but companies typically execute a pilot project when a technology is not economically efficient or feasible. A company without a long-term perspective for its business can buy time by realizing pilot projects (Lebelhuber & Greiling, 2022). Specific developments that may not effectively reduce carbon emissions but can delay substantive climate action include carbon offsetting (Guix et al., 2022), sustainable fuel technology (Dodd & Yengin, 2021), and carbon credits (Rajão & Marcolino, 2016).

### Managing Intermediaries: Certification and Metrics Greenwashing

In addition to the plethora of new stakeholders that populate the greenwashing landscape and emergent research, firms are now increasingly tasked with engaging with and managing a new player: information intermediaries. We consider intermediaries to be any of a variety of government, NGO, media, and standards and certification bodies that seek to scrutinize and endorse, where warranted, firms’ social and environmental claims. These entities typically aim to increase the transparency and reliability of information about corporate behavior. However, as these intermediaries become more widely known and accepted, corporate greenwashing efforts are extending to manipulating the information they produce. To date, the literature has largely focused on greenwashing that seeks to manipulate certifications and metrics, although we expect similar efforts are underway with regard to government, NGO, and media intermediaries.

A number of popular commentators have noted the growing prevalence of greenwashing in the very tools intended to address it, including in rapidly growing ESG and net zero commitments (Black et al., 2021; Quinson, 2021). Even as interest grows in certifications and ecolabels as a means to prevent greenwashing, these accounts suggest that measurement tools themselves have become a target and tool of greenwashers (e.g., (Berg et al., 2022; Fabrizio & Kim, 2019; Montgomery & Lyon, 2021). The literature is growing rapidly in this area, with several recent papers noting that firms may be using the legitimacy and symbolic cover of a label to underperform on their environmental promises, or at least to underperform what consumers may expect from certified firms, revealing that even well-intentioned labels can facilitate greenwashing. For example, Flowers et al. (2020), studying LEED certification, illustrate that firms are able to accrue points without actually reducing emissions, the goal of the program. Liute and De Giacomo (2022), studying B Corps, find that firms underperform or fail to contribute to environmental reduction goals. A series of papers examining ISO 14001 standards have similar findings (Aravind & Christmann, 2011; Garrido et al., 2020; Heras-Saizarbitoria et al., 2020; Wagner, 2022), including the use of certification as a symbolic measure, few emissions reductions, and no positive effects on environmental metrics. The growing greenwashing in certifications may be one reason why firms choose not to tout their certifications, and to brownwash (Carlos & Lewis, 2018; Montgomery & Robertson, 2022).

Of course, a fundamental problem in fields with substantial social and environmental impacts is that it is difficult for consumers and other stakeholders to verify the impacts of particular products, rendering the field “opaque.” The products exchanged in such fields are called “credence goods” in economics, since the consumer generally has to take claims about product impact on faith. It is difficult for even highly-trained observers to ascertain the connection between means (improved production processes) and ends (social and environmental impacts) in such fields, which can facilitate a decoupling of the two (Bromley & Powell, 2012). Metrics and certifications designed with the best of intentions may be unable to specify accurately the set of practices required to achieve a goal such as the sustainability of a fishery, reducing the effectiveness of the standard and rendering it a potential vehicle for greenwash (Wijen, 2014; Wijen & Chiroleu-Assouline, 2019). In addition, it is widely recognized that certifications with weak enforcement may facilitate symbolic participation, decoupling, or greenwashing, especially among firms that are late joiners (Delmas & Montes-Sancho, 2010; King & Lenox, 2000; Wijen, 2014).

Moreover, as metrics and certifications proliferate, it is common that consumers become confused about the meaning of the various alternatives (Harbaugh et al., 2011; Heyes et al., 2020), exacerbating the opacity of the field. This problem is manifest in the enormous proliferation of ESG claims, rating schemes, and investment vehicles in recent years, which makes it difficult for observers to determine what each one means and how they differ from one another. In fact, research shows that different ESG rating agencies vary enormously in their evaluations of the same firm, raising concerns that the net result of the rating game is merely “aggregate confusion” (Berg et al., 2022). This problem reflects an important issue within the larger world of environmental standards and certification programs, of which there were 459 globally as of the end of 2020, far more than most consumers can make sense of (Ecolableindex.com). The resulting confusion can function as a form of greenwashing, even if standards are not intentionally designed to obfuscate (Lyon & Montgomery, 2015). Practitioners have long known that labels can be intentionally misleading, as indicated by TerraChoice’s “Seven Sins of Greenwashing,” which include “worshipping false labels” (TerraChoice, 2009). The research cited above is revealing that even well-intentioned labels can inadvertently facilitate greenwashing.

Future research could draw on the significant emerging literature on metrics and reporting to further tease out the mechanisms by which greenwashing is created through labels. Research might expand on interesting forays into: how numerous labels might confuse consumers; how legitimacy for new labels is achieved; how metrics are lowered or altered to allow firms with questionable green credentials to join; and how legitimate or well-meaning businesses are engaging in such labels, if they are. Turning to impacts, scholars also have little understanding of how already sustainable firms are being affected by the emergence of new labels, how they react, whether their attempts to either call out or join such labels (“raise the bar”) are impactful, and what are the environmental impacts of greenwash via label proliferation.

### Managing New Issues

The greenwashing literature has also inspired scholars to take a closer look at companies’ management of other issues. The suffix “-washing” has become synonymous with any activities by corporations that are intentionally misleading or fall behind on expressed values. The two most salient examples are the recent problematization of lobbying activities, and the recent trend of corporate communications that are allegedly “woke” but potentially hypocritical.

#### Corporate Political Action and Government

In recent years, several prominent sustainability academics (Lyon et al., 2018), as well as numerous practitioners (e.g., World Benchmarking Alliance, InfluenceMap), have begun to call for more attention to the political activities and lobbying efforts of corporations, and whether these align with companies’ publicly stated social and environmental positions. Anecdotal evidence suggests that misalignment between these two strands of corporate activity is widespread (Delmas & Lyon, 2018), and we see this as an important emerging form of greenwashing. Academic research is just beginning to address the issue of (mis)alignment. Favotto and Kollman (2021) present one of the first academic studies in this area, and find evidence that misalignment may be less prevalent than expected, since U.K. firms that undertake more CSR activities tend to offer more substantive and scientifically grounded public testimony in Parliament.

This emerging stream of the greenwashing literature highlights the variety and complexity of interactions between firms and governments. Prior to the emergence of this new stream, the regulator typically appeared as a watchdog and moderator of the temptation to greenwash, or as a mediator of activists’ efforts against greenwashing (e.g., Kaplan & Perez, 2021; Marquis et al., 2016; Roesler, 2017; Rotman et al., 2020). In that view, firms’ communications of their environmental impacts do not differentiate between the government and other actors (Crilly et al., 2016), and firms may respond to regulatory pressures—just like they respond to activists’ demands–with greenwashing (Setia et al., 2015; Wedari et al., 2021). Furthermore, firms may tout their legally mandated emissions reductions or transparency improvements as evidence of their own virtue, turning regulatory mandates into a greenwashing tool (e.g., Leung & Snell, 2021; see also Sin of Irrelevance in TerraChoice, 2009).

In contrast, recent research notes the exceptionally powerful role of the government, which controls access to many natural resources and the rules around pollution. The special role of the government is in the spotlight especially after Lyon et al. (2018) argued that a politically responsible firm should ensure that its lobbying activities are in line with any self-proclaimed green goals, highlighting a novel form of greenwashing by firms who do not. If the lobbying efforts are not made public, the firm can maintain its green image despite deploying vast sums to block public policy from emerging. For instance, the seven largest U.S. oil and gas companies have actively lobbied politicians to allow drilling in the Arctic National Wildlife Refuge—a move that clearly contradicts their expressed commitment to biodiversity protection (Cho et al., 2018). Large companies like ExxonMobil, Chevron, and Koch Industries are so brazen in their lobbying that some authors suggest their commitments to sustainability may need a new term, “green gilding” rather than greenwashing (Parafiniuk & Smith, 2019). Because of this complex and interdependent relationship between government and business, a firm that is deeply embedded in the local community may maintain public support even while destroying the local environment (Brock & Dunlap, 2018).

In addition to being the target of greenwashing, government can also be complicit in or engage in greenwashing. When economic growth is a more important political outcome than environmental protection, regional governments may be less eager to implement national environmental policy: governance becomes green on paper and brown in practice (Alon-Barkat, 2020; R. Wang, Wijen, et al., 2018). In addition, government agencies risk enabling greenwashing if they enact legislation on environmental disclosure without also enforcing environmental performance improvements (R. Huang & Chen, 2015). Governments can also engage in more tangible forms of greenwashing. For example, many city carbon neutrality pledges involve the use of biomass energy, which unlike wind and solar, entails significant environmental issues, including potentially negative climate impacts from air pollution (Harjanne & Korhonen, 2019).

#### Wokewashing, Genderwashing, Diversitywashing, and More

Similar practices to greenwashing have long been noted in the literature. These include “pinkwashing” for breast cancer branded products, “SDGwashing” for the UN sustainable development goals (Ferrón-Vílchez et al., 2022), and “bluewashing,” which has been used both to apply to firms wrapping themselves in United Nations language and to the use of water-related advertising and miscommunication. However, in response to recent movements around #MeToo and Black Lives Matter, and a renewed interest in social justice and inequality in management literature (e.g., Amis et al., 2021; Montgomery & Dacin, 2020, 2021), these claims have exploded, often under the colloquial and intentionally ironic term “wokewashing” (Sobande, 2019), that underlines the deceptive and inauthentic nature of the practice. Similarly, the use by firms of “symbolic civil rights” (Buchter, 2021; Edelman, 2016) has been noted in the literature as has the use of CSR and greenwashing around gender equity and diversity goals in publicity by firms, termed “femvertizing” (Sterbenk et al., 2022) or “gender washing” (Walters, 2022).

Although this emerging research spans numerous related disciplines and terms, and a full discussion and analysis are beyond the scope of the current review, we note that these practices and emerging terms are closely related to greenwashing. Future research might explore how the tactics used are similar or different given the social context and the underlying complexities around inequality and diversity. Likewise, insights from the more mature greenwashing literature could be used to inform these growing areas of study, drawing together richer theory on corporate miscommunications at their intersection.

## Greenwashing 3.0: Creation of Narratives About the Future

As shown in Figure 1 (lower right panel), there has been a recent explosion of news stories involving greenwash, from about 700 stories in 2020 to over 2,300 in 2021, roughly double the number of stories in 2009, the previous record year. Academic research moves at a much slower pace than the news, but it is reasonable to expect that the phenomena driving this resurgence of interest will set the agenda for greenwashing research over the next few years. Two broad themes appear to be at the heart of the renewed interest in greenwash: investing with a focus on ESG criteria, and corporate “net zero” commitments.

### Greenwashing and ESG Commitments

One of the most prominent voices in the ESG investing debate is that of Tariq Fancy, former head of sustainable investing for Blackrock, who has publicly argued that the ESG investing industry is engaging in an elaborate greenwashing effort that has little or no impact and merely distracts the public from the need for strong government action to address climate change.^2^ In contrast, some right-wing politicians fear that ESG investing is so impactful that it threatens the very future of fossil fuels and the campaign contributions that come along with them (Gelles, 2022). ESG investing offers the attractive hope that investors can make more money by investing in things that save the planet, whistling past the realization that we would not be facing a climate crisis were this hope actually true in general. For the most part, academic research provides little support for the notion that markets will self-correct and automatically solve environmental problems without government action (Margolis & Walsh, 2003), although recent work using regression discontinuity methods suggests that shareholder CSR proposals indeed seem to enhance financial performance (Flammer, 2015).

Whether broader ESG ratings truly signal better future financial performance remains controversial. Accounting research by M. Khan et al. (2016) suggested that firms earned higher returns if they received high ESG ratings on “material” dimensions of non-financial performance (defined, somewhat circularly, as those dimensions that matter for financial performance). However, attempts to replicate their findings suggest that these results are extremely fragile and may simply be a statistical artifact (King & Berchicci, 2022). Research using a greenwashing lens has only begun to explore the performance of ESG investing. Early research in this space by Boiral and Henri (2017), sampling ESG reports rated A and A+ by the Global Reporting Initiative, has found that firms re-use the same language for their future commitments again and again over the years in order to greenwash, delay, and not comply with reporting requirements. Barrymore (2021) also finds that investors’ ESG preferences correlate with symbolic corporate policies but not actual outcomes, suggestive of greenwashing, and the extent of greenwashing increases for firms with greater ESG rating disagreement. Since recent work documents the enormous extent of disagreement between different ESG ratings (Berg et al., 2022), and since ESG ratings firms have strong incentives to keep their “secret sauce” proprietary, it is unlikely that ESG rating harmonization will occur any time soon. Thus, research on ESG ratings as a form of greenwash is likely to continue to increase in coming years.

Whether research on ESG investing will expose entirely new mechanisms of greenwashing is unclear. Claims based on limited evidence, like many ESG investing claims, are a staple of traditional greenwashing. However, recent financial research on so-called “distracted investors” (institutional shareholders who experience a shock to one portion of their portfolios and thus pay less attention to other portions) may provide a useful perspective on greenwashing. Kempf et al. (2017) find that firms with “distracted” shareholders are more likely to announce diversifying acquisitions that destroy firm value, grant opportunistically timed CEO stock options, and cut dividends, and are less likely to fire their CEO for bad performance. It seems natural to expect that firms are more likely to greenwash when investors are distracted. Moreover, large diversified firms may be able to create distractions intentionally in one division of the firm so as to divert attention from greenwashing in another division. W. Huang et al. (2021) take a complementary perspective, focusing on measures of attention to ESG issues on the part of a firm’s employees and investors; they find that such attention positively correlates with a number of measures of corporate ESG performance.

### Greenwashing and Net Zero Commitments

The second topic that has attracted a slew of greenwashing accusations in the popular press is corporate net zero claims. Many such claims appear to have their origins in the Trump Administration’s repudiation of the Paris Agreement, which spawned a number of governance responses from sub-national levels of government as well as private sector initiatives. One prominent example is “We Are Still In,” which claimed 2,301 private sector participants as of September, 2022. Launched on June 5, 2017, the initiative explicitly called out the Trump Administration’s backsliding on climate action, and claims to be the “largest cross section of the American economy yet assembled in pursuit of climate action.”^3^ Corporate net zero pledges have proliferated since that time, as have efforts to track and evaluate them. According to Net Zero Tracker, as of June 13, 2022 “More than one third (702) of the world’s largest publicly traded companies now have net zero targets, up from one fifth (417) in December 2020. But 65% (456/702) of corporate targets do not yet meet minimum procedural reporting standards.”^4^ A more recent initiative, launched June 7, 2022, is the Claims Code of Practice launched by the Voluntary Carbon Markets Integrity Initiative, which is backed by the British government. It seeks to help investors determine whether claims made by companies using carbon offsets are credible.^5^

Not surprisingly, environmental groups have expressed much “skepticism about these corporate targets and concerns that they are a new form of greenwashing. Critics have been especially concerned about corporate net zero targets depending heavily on carbon offsets, rather than a business taking rapid action to decarbonize its own value chain where accountability and influence are highest.”^6^ The World Resources Institute has developed three criteria to help distinguish legitimate claims from greenwash,^7^ while Columbia’s Center for Sustainable Investment offers a more detailed set of 8 criteria for assessing greenwashing in corporate net zero claims.^8^

Because net zero commitments are by nature long-term promises, rather than verifiable statements about current performance, they open up a whole new world of greenwashing mechanisms, which we label “futurewashing.” The emerging criteria for evaluating greenwashing point to mechanisms for futurewashing have not yet received much academic attention, with a few exceptions that note the use of future commitments as a mechanism (Talbot & Boiral, 2018). First, corporate claims may fail to incorporate short-term targets, making it impossible for stakeholders to assess their validity. Second, long-term targets may rely on approaches such as carbon offsets or sequestration, whose feasibility or reliability is uncertain and which many groups consider incompatible with scientific knowledge about what will be required to achieve aggregate net zero emissions.^9^ Third, the long-term nature of such commitments, and the fact that they are not legally binding, makes them highly vulnerable to future renegotiation and retreat.

A small body of emerging academic research is already beginning to explore these new modes of greenwashing, much of it still in the publication process as of this writing. Callery and Kim (2020) analyze CDP data from 2011 to 2019 and compile a data set of 24,098 individual carbon targets from 2,438 firms. They find that corporate claims of target attainment frequently contradict calculations of actual target attainment based on disclosed carbon emissions; moreover, firms regularly alter target parameters from year to year with the adjusted targets becoming less aggressive over time. More broadly, Bolton and Kacperczyk (2022) study 17,385 companies over the period 2011 to 2019, finding that the companies that make commitments tend to be companies with lower emissions, and that although these companies do subsequently further reduce their emissions, the effect of these initiatives on overall emissions of publicly traded companies has been small. Overall, these early research efforts suggest, worrisomely, that voluntary corporate commitments may be unlikely to produce substantial progress toward a net zero future. Clearly further research is needed in this area.

## A Proposed Model of Corporate Miscommunication

Based on our discussion above, it is evident that the literature on greenwash has matured to the point that the outlines of a general theory for understanding corporate communication and miscommunication are becoming visible. In this section we attempt to sketch the key elements of such a theory, and how they are connected. The centerpieces of the emerging theory are (a) the nature of the issue at stake, (b) the multiple stakeholders to whom the firm communicates on that issue, (c) the intermediaries who scrutinize and either endorse or criticize the firm’s messages, (d) the cognitive biases and attentiveness of each stakeholder, and (e) the social network within which the stakeholders are embedded. These elements are illustrated in Figure 4, which depicts the evolution of the emerging model of greenwashing that we seek to capture here.

Greenwashing 1.0 incorporated only the first three of the above elements, and even then only in a limited fashion. It tended to focus on a single issue that was of interest to only one stakeholder group (usually consumers), and to include at most one intermediary (usually an NGO) that could provide independent scrutiny of the firm’s messages. Greenwashing 2.0 incorporates more complex issues that might be of interest to multiple stakeholders, begins to identify the cognitive limitations of the various stakeholders, and recognizes the potential for multiple intermediaries to scrutinize the firm’s messaging. It also allows for the possibility that the firm depends upon upstream suppliers for some of its own information. Greenwashing 3.0 reflects the increasing role of the financial industry in sustainability communications. Investors emerge as a key target of messaging, with the potential to reward or punish the firm through their allocation of capital, and with their information influenced by auditing and ratings firms, as well as the other intermediaries identified in Greenwashing 2.0. In the remainder of this section we explain the elements of the model in more detail.

### The Issue

Although it may seem to go without saying, the nature of the issue at stake is a crucial factor in greenwashing behavior. Key dimensions of issues include the spatial and temporal distribution of external impacts across individuals, the demographic characteristics of those affected (especially their wealth and political power), the impact of the issue on the firm’s bottom line, and stakeholder judgments regarding the legitimacy of the issue and the firm. All of these dimensions of the issue shape the set of stakeholders that respond to the firm’s messaging, and the strength of their responses.

### Multiple Stakeholders

The incentive to greenwash depends upon the pressure firms receive from stakeholders regarding the issue. The literature argues that firms attend to stakeholders possessing some combination of power, legitimacy and urgency (Mitchell et al., 1997). This will differ with each particular issue, of course, as well as with other factors affecting particular stakeholders at a particular time. For example, investors may be less receptive to green initiatives when the firm is performing poorly from a financial perspective, and regulators may be more receptive to green initiatives when a firm is seeking a permit for expansion of a polluting facility (E.-H. Kim & Lyon, 2015).

### Intermediaries

Firms are often scrutinized by external groups such as NGOs, the government, the media, certifying bodies, and rating agencies. These intermediaries may issue their own communications to stakeholders, either endorsing the firm’s claims, criticizing them or providing independent assessments (Bagnoli & Watts, 2017; Berg et al., 2022). These intermediaries have been recognized as crucial challenges of greenwash even in Greenwashing 1.0 (Lyon & Maxwell, 2011; Lyon & Montgomery, 2013), but more recent work allows for multiple intermediaries and considers strategic responses by firms to the fact of being evaluated. For example, certain stakeholders are likely to attend to communications from certain intermediaries, while others will not (Bagnoli & Watts, 2017). Depending upon the sophistication of the intermediaries, and their importance to key stakeholders, the firm may decide to devote effort to “gaming” the communications of the intermediaries, which becomes a key part of the research activity in Greenwashing 2.0 (Fabrizio & Kim, 2019) and 3.0 (Callery & Kim, 2020).

### Cognitive Biases

Research shows that stakeholder attentiveness and cognition are crucial in determining how the stakeholder responds to information. Early research on “symbolic management” found that when firms announce they are going to offer CEO stock options, investors pay more attention to how the announcement is framed than to whether the firm actually follows through with granting the options (Westphal & Zajac, 1998). This lack of attention to detail on the part of people with significant amounts of money at stake remains a striking and important reminder that humans have cognitive limitations, and minds that are often distracted by other issues. Recent work in finance finds that firms achieve lower CSR ratings when institutional investors are “distracted” by exogenous shocks in other parts of their portfolios (T. Chen, Dong, & Lin, 2020). If even institutional investors are easily distracted, surely so are other stakeholders with less at stake. Thus, stakeholder attentiveness is a critical factor in a theory of greenwash and is an important factor in determining whether an issue is “salient” to a given stakeholder or not.^10^ Other dimensions of cognition are also important in defining salience. An employee with training in environmental science may react much more negatively to corporate greenwash than an employee without that training (Robertson et al., 2023). Partisan political leanings shape how people respond to claims about the importance of addressing climate change (Dunlap et al., 2016). People also differ widely in how much trust they place in corporations. As a result, some stakeholders may take corporate claims at face value, some will reject them, and some will evaluate them rationally based on all the available evidence (Bagnoli & Watts, 2017).

### Social Networks

Finally, a theory of greenwash must comprehend the social network within which the firm’s stakeholders operate. The firm may wish to target different messages to different audiences, and if those messages appear to be at odds with one another, then stakeholders could become angry and punish the firm if they receive multiple conflicting messages, much as the NGO punishes the firm for greenwash in the model of Lyon and Maxwell (2011). In order for the firm to successfully target different stakeholders with conflicting messages, it must be able to partition the audiences and (largely) prevent the different stakeholder groups from sharing their messages across partitions. Targeting has become much easier in the era of social media, as advertisers can target extremely narrow demographic groups. But targeting fails if messages intended for one stakeholder group are forwarded to members of other stakeholder groups that object to them. Indeed, part of the reason authors have anticipated the end of greenwash (Bowen, 2014; Lyon & Montgomery, 2015) was that they expected the internet to enable vigilant consumers to rapidly call out corporate greenwash (Lyon & Montgomery, 2013). However, recent research on social networks finds that they demonstrate a remarkable amount of “homophily,” that is, the tendency to communicate primarily with members of one’s own group. Models of social networks with homophily show that information can be very slow to diffuse throughout society when differing groups seldom communicate beyond their own bubbles (Golub & Jackson, 2012). This delay in convergence of beliefs can be a serious problem when it causes public policy to be deferred on critical issues such as climate change. However, it offers a powerful opportunity to companies that wish to partition their stakeholders.

Combining these basic primitives of the model, we can sketch out graphically the emerging structure of the theory, as in Greenwashing 2.0 & 3.0 in Figure 4. Suppose the firm has four stakeholders, two skeptical and two trusting, and can deliver messages to each of them. A skeptical stakeholder only believes corporate messages that are verifiable and that she has taken the trouble to verify; a trusting stakeholder believes whatever the firm tells him. The stakeholders are part of a larger social network, and engage in discourse with other stakeholders. However, stakeholders have a preference for engaging with other members of their own group more often than with members of other groups. If, for example, trusting stakeholders only communicate with other trusting stakeholders, then it is easy for the firm to greenwash its communications to these stakeholders, even as it provides more complete and accurate information to skeptical stakeholders. Of course, homophily is often incomplete, and there is likely to be some communication between skeptical and trusting stakeholders, making the evolution of beliefs a complex but fascinating process.

Finally, the various stakeholder groups offer rewards or punishments to the firm based on the information they receive from other stakeholders and from intermediaries. The firm then designs a communication strategy to maximize the rewards it receives from stakeholders and minimize the punishments it absorbs. The firm will be more likely to greenwash or brownwash when its stakeholder groups are trusting and highly isolated from one another, in which case they can be given misaligned messages with little chance of being detected. The firm will be less likely to greenwash or brownwash when all stakeholder groups are in communication with one another.

The direction of information bias, that is, whether the firm prefers to greenwash or brownwash a given stakeholder, depends upon whether the stakeholder views a green initiative positively or negatively, and how much information the stakeholder receives from other sources. The extent of information bias depends upon the extent to which the stakeholder trusts the firm as a source of information, as well as the extent to which the stakeholder relies solely upon the firm for information. Because of the dynamic nature of communication between groups, the emerging model of greenwash plays out over time, and the firm’s communication strategy will evolve over time. Indeed, it is in part exactly this evolution of strategy that we are tracing out throughout the course of this review.

## Conclusion

As we look over the last decades of greenwashing research, two things are apparent: First, the scholarly community has done good research, explored new mechanisms and teased out existing ones, and added novel stakeholders, actors, relationships, and contexts. The “means” of greenwash have been well but not exhaustively explored. Second, greenwashing shows no signs of abating despite our excellent research. The “ends” continue to elude even a passionate and determined cadre of academics.

Any scholar who cares about practical impact, and there are many of us, must wonder where we went wrong. Have we perhaps given too much attention to the phenomenon, putting ideas in the minds of nefarious marketers and managers everywhere? Were we simply not aware of how significant the problem was until we began to scratch the surface? Has our work failed to cross the infamous translation gap to practitioners, not offering comprehensible and practical solutions to these problems to well-meaning executives, activists, NGOs, watchdogs, and policymakers who might end it? Or has the problem simply gotten worse as stakeholders press companies more than ever to be green? Although the answer no doubt lies somewhere at the intersection of these causes, and we may never fully know, we should consider each of these as we design and conduct future research.

In our opinion, the most impactful research going forward will be that which gets to the crux of when, why, and how corporations greenwash, with the intent being not to simply show that it is happening, but to consider what tools can stop it. Testing the roles of transparency, stakeholder activism, government policy, and more, with clear and actionable solutions, while connecting with and supporting businesses and other practitioners who seek to avoid greenwashing, will lead the way. It is our hope that in another 8 long years, scholars—other than us!—will be writing a review detailing how greenwashing research tackled these problems and has begun to finally stifle corporate miscommunication once and for all.

## Notes

1.
https://charteredabs.org/academic-journal-guide-2021/

2.
https://www.economist.com/by-invitation/2021/11/04/tariq-fancy-on-the-failure-of-green-investing-and-the-need-for-state-action

3.
https://www.wearestillin.com/we-are-still-declaration

4.
https://zerotracker.net/insights/pr-net-zero-stocktake-2022

5.
https://www.reuters.com/business/environment/initiative-launched-rank-corporate-climate-claims-using-carbon-offsets-2022-06-06/

6.
https://www.wri.org/insights/ways-companies-credible-net-zero-targets

7. The WRI criteria are: 1) Set Near-Term Science-Based Targets, 2) Set Long-Term Science-Based Net Zero Targets, and 3) Invest in Cutting Emissions beyond the Value Chain. https://www.wri.org/insights/ways-companies-credible-net-zero-targets

8. The WRI criteria are: The Columbia criteria are: 1. Does the company have short- and medium-term targets?, 2. Are the targets based on absolute emissions or on emissions intensity?, 3. Are the targets aligned with science-based climate goals?, 4. Does the company’s net-zero target include scope 3 GHG emissions?, 5. Does the company consider targets in planning for capital expenditures (CAPEX), use an internal carbon price, or both?, 6. Does the company rely on carbon offsets, carbon capture and sequestration (CCS), or both?, 7. Does the company’s governance structure incentivize climate action?, 8. Does the company have a policy not to lobby against climate policy? https://ccsi.columbia.edu/news/corporate-net-zero-pledges-bad-and-ugly

9. Other aspects of greenwashing that have been identified in corporate claims fall under existing criteria. For example, they may engage in selective disclosure by covering an unduly restricted scope of emissions (often only including what are known as Scope 1 emissions, those coming directly from corporate smokestacks, but not Scope 2 emissions, coming from purchased electricity, or Scope 3 emissions, coming from the use phase of products). In addition, corporate political actions may fail to support policies that would accelerate climate action, which can also be considered a form of selective disclosure if the firm hides its political activities using dark money or front groups.

10. From a cognitive perspective, the firm’s managers also respond to particular issues based on their “salience,” that is, “the degree to which a stakeholder issue resonates with and is prioritized by management.” (Bundy et al., 2013, p. 353). Although this definition is foggy (how is “resonance” measured?) and borders on the tautological (“management responds to issues that are prioritized by management”), it does usefully point to the importance of cognitive factors in how managers approach an issue.
